# Case Report: Simnotrelvir/Ritonavir are effective in shortening the course of prolonged SARS-CoV-2 infection during anti-CD20 maintenance therapy in patients with follicular lymphoma

**DOI:** 10.3389/fonc.2025.1583932

**Published:** 2025-08-13

**Authors:** Jing Yang, Shengke Tu, Hui Peng, Yuanyuan Peng, Min Li, Kui Song

**Affiliations:** ^1^ Department of Hematology, The First Affiliated Hospital of Jishou University, Jishou, Hunan, China; ^2^ Department of Pharmacy, The First Affiliated Hospital of Jishou University, Jishou, Hunan, China

**Keywords:** follicular lymphoma, COVID19, anti-CD20, SARS-CoV-2, infection, Simnotrelvir/Ritonavir

## Abstract

Patients with hematologic malignancy had the possibility of persistent infection with COVID-19, particularly those who received anti-CD20 monoclonal antibody therapy for lymphoma, regardless of vaccination status. We report two cases of follicular lymphoma (FL) infected with Omicron virus that could not be confirmed by routine SARS-CoV-2 tests during maintenance therapy with an anti-CD20 agent, obinutuzumab or rituximab. In addition to immunomodulatory drugs, both of them took Simnotrelvir/Ritonavir to effectively alleviate the symptoms. The aim is to highlight the complexity of SARS-CoV-2 infection and to discuss why it is easy to be infected with COVID-19 for a long time in this fragile patient population and the response to such patients with unexplained respiratory symptoms during maintenance therapy. Patients with hematologic malignancies treated with anti-CD20 agent are at considerable risk of a prolonged disease course and recurrence of COVID-19. Specialized prevention, diagnostic and therapeutic strategies should be developed for this group of patients.

## Introduction

Omicron was first detected in South Africa on November 9, 2021, and the World Health Organization defined it as the fifth variant of concern of SARS-CoV-2, saying that its overall global risk assessment is extremely high and may spread globally (3). According to early data, Omicron is more infectious and reduces the vaccine effect. At the same time, the severity also decreases (4). However, infection in immunosuppressed patients and those undergoing chemotherapy are associated with a high risk of severe disease (5).

In patients with B-cell lymphoma, the serological response to COVID-19 vaccine is impaired due to immunodeficiency, especially in those with follicular lymphoma who have recently been treated with anti-CD 20 monoclonal antibodies, because anti-CD20 antibodies bind to the CD20 antigen of B cells to induce complementarity or antibody-dependent cytotoxicity, which leads to antitumor effects (1, 2). Moreover, in this process, B cell depletion persists after anti-CD20 treatment (6).

In patients with B-cell lymphoma, serologic response to the COVID-19 vaccine is compromised due to immunodeficiency, especially in patients with FL who have been recently treated with anti-CD 20 monoclonal antibody, because the anti-CD20 antibody binds to the CD20 antigen of the B-cells and induces complement- or antibody-dependent cytotoxicity, which leads to antitumor effects. And in this process, B cell depletion persists after anti-CD20 treatment.

We report herein two cases of patients receiving Bendamustine associated with an anti-CD20 agent, Obinutuzumab or rituximab for FL. Both with respiratory symptoms and Serological antibody detection and nasopharyngeal swab were negative to SARS-CoV-2 by RT-PCR during maintenance treatment (**Table 1**). Monoclonal antibodies (mAB) are homogeneous antibodies derived from a single B-cell clone, characterized by their high specificity in targeting defined antigenic epitopes (e.g., viral surface proteins or tumor-associated antigens). Rituximab is a chimeric (human/murine) anti-CD20 mAB causing rapid and persistent depletion of CD20^+^B cells, while Obinutuzumab is a glycoengineered type II anti-CD20 monoclonal antibody that has lower complement-dependent cytotoxicity than rituximab but greater antibody-dependent cellular cytotoxicity and phagocytosis and greater direct B-cell killing effects (7). Anti-CD20 mAB + chemotherapy is preferentially recommended for patients with an indication for treatment at diagnosis. Anti-CD20 mAB was selected with either Obinutuzumab (G) or rituximab (R) and chemotherapy with Bendamustine (B)/CHOP regimen/CVP regimen. The results of StiL and BRIGHT studies showed that BR regimen was superior to R-CVP/CHOP regimen, but the incidence of infection was high and attention should be paid to prophylaxis (8, 9).

**Table 1:** Baseline characteristics of patients and data for each respiratory symptoms episode.

Baseline	Case 1						Case 2			
Age	58						59			
Sex	M						F			
Autoimmune disease	No						No			
ICT First line of ICT	R-G						R-B			
Second line of ICT	N/A						N/A			
Date last anti-CD20	<6 month						<6 month			
Symptoms	EP1	EP2	EP3	EP4	EP5	EP6	EP1	EP2	EP3	EP4
Hypogammaglobulinemia	No	No	N/A	No	No	No	N/A	Yes	Yes	Yes
Days from symptom onset	2	4	15	10	12	6	4	20+	40+	2
Fever	Yes	No	No	No	Yes	Yes	Yes	Yes	Yes	No
Pharyngalgia/cough/expectoration	No	Yes	Yes	Yes	Yes	Yes	Yes	Yes	Yes	Yes
Pneumonia	No	No	No	No	Yes	YES	No	Yes	Yes	Yes
NEU%	85.30	73.40	44.70	38.00	74.10	64.40	71.20	59.80	63.80	79.40
LYM%	3.00	10.70	16.60	27.60	14.60	21.20	7.00	21.20	20.70	9.20
Platelets, ×10^9^/ L	114	96	128	169	120	221	100	147	253	153
CRP, mg/L	23.30	3.80	5.23	4.12	29.14	N/A	22.94	11.72	15.8	2.77
D-dimer, ng/mL	N/A	N/A	N/A	N/A	N/A	N/A	N/A	0.51	N/A	N/A
RT-PCR	NEG	NEG	NEG	NEG	NEG	NEG	N/A	POS	NEG	NEG
SARS-CoV-2 IgG antibodies	N/A	N/A	N/A	N/A	NEG	ENG	N/A	N/A	N/A	NEG

F, female; M, male; ICT, immunochemotherapy; R-G, bendamustine- obinutuzumab; R-B, bendamustine-rituximab; N/A, not applicable EP1, first episode; EP2, second episode; EP3, third episode; hypogammaglobulinemia, IgG, IgA , IgM level lower than normal ; NEU%, Percentage of the neutrophils; LYM%, Percentage of the lymphocytes;CRP, C-reactive protein; RT-PCR, reverse transcriptase polymerase chain reaction; NEG, negative; POS, positive.

Simnotrelvir/Ritonavir (XIANNUOXIN) is a co-packaged combination drug consisting of simnotrelvir and ritonavir, both ingredients are oral tablets. Simnotrelvir (SIM0417) is an oral antiviral agent targeting the 3-chymotrypsin-like (3CL) protease, which is essential for SARS-CoV-2 viral replication. The 3CL protease, also known as the main protease (Mpro), is a three-domain cysteine protease (10). It is recommended that simnotrelvir/ritonavir is administered every 12 h for 5 days according to the package insert of XIANNUOXIN. Simnotrelvir, as a derivative of nirmatrelvir, has a chemical structure and pharmacological activity similar to those of nirmatrelvir. It suppresses SARS-CoV-2 Mpro activity by binding to the active site of SARS-CoV-2 Mpro by means of a reversible covalent method, and it renders proteins unable to process polyprotein precursors, leading to inhibition of viral replication (11, 12). Ritonavir is a potent CYP3A inhibitor as well as an oral antiretroviral agent for the treatment of the human immunodeficiency virus (HIV) (11). Ritonavir retards the metabolism of simnotrelvir by inhibiting the CYP34A enzyme, thereby improving the bioavailability of simnotrelvir and enhancing its antiviral activity (13).

## Case report 1

**Figure 1:**
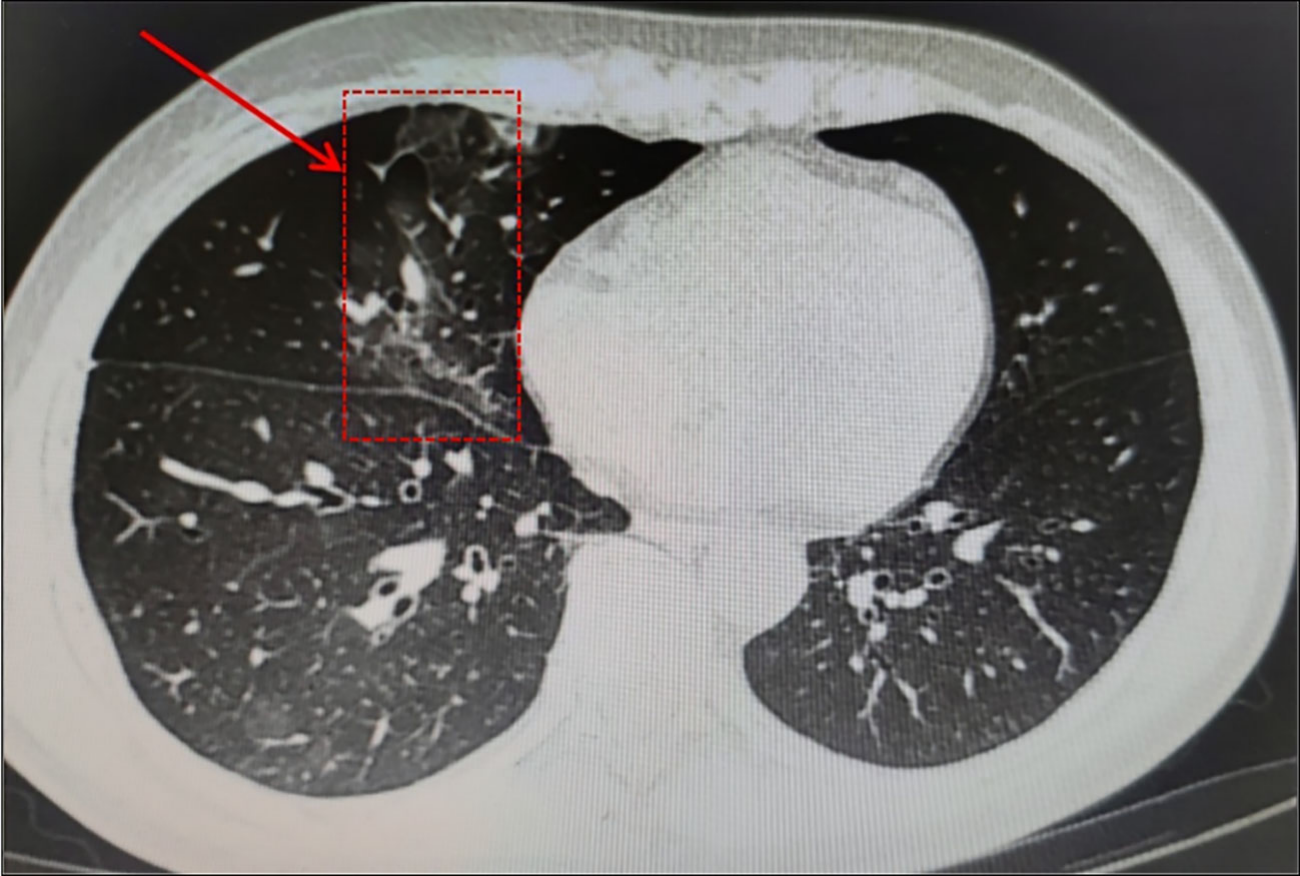
Right middle and lower lung infection.

A 58-year-old male patient, beginning with multiple swollen lymph nodes in the abdominal cavity, was diagnosed with stage IV-A FL in January 2022 and was classified as intermediate risk according to FLIPI (2 points). In February 2022, we had a lymphoma Multi-Disciplinary Treatment(MDT) discussion, and he started the first cycle of Obinutuzumab chemo-immunotherapy (1000mg d1 8). He received COVID-19 vaccine before the chemotherapy. Following three cycles, in April 2022, he began taking Bendamustine (150mg d 2 3) and Obinutuzumab (1000mg d 1). He started to cough with phlegm and have a sore throat during this round of treatment. A nasopharyngeal swab was negative to SARS-CoV-2 by RT-PCR. It is our belief that he suffered from acute bronchitis, and his symptoms improved after administering cefuroxime sodium. In February 2023, he was switched back to Obinutuzumab monotherapy maintenance therapy. Following the administration of Obinutuzumab on the third day of hospitalization in September 2023, he began to cough with white mucous sputum. On the fourth day, he experienced a fever and on the fifth day, he coughed up thick yellow sputum. A computer tomography scan (CT) was performed as monitoring of his right lower lung infection. A subsequent CT scan three days later revealed a more infected right lower lung than in the anterior and all tests for influenza A/B virus antigen, serological testing, sputum culture and identification, and RT-PCR were negative. His fever remained despite our prescription of ceftazidime, so we switched to piperacillin sodium tazobactam sodium to treat his illness. He continued to be feverish, but he insisted on leaving the hospital. He was readmitted after a fortnight because of a persistent fever, cough, and expectoration. All microbiologic tests for respiratory pathogens were negative. A new CT scan was performed showing lung infection (**Figure 1**) is worse than before. Other infections, reactivation of autoimmune disease, and recurrence of lymphoma were ruled out. A bronchoscopy was performed, and a bronchoalveolar lavage (BAL) sample tested positive for SARS-CoV-2 (Omicron-XBB.1:21977:>1.0E+6). He rapidly dropped his fever after we gave him Lianhua Qingwen granules and the anti-SARS-CoV-2 drug Simnotrelvir/Ritonavir.

## Case report 2

**Figure 2:**
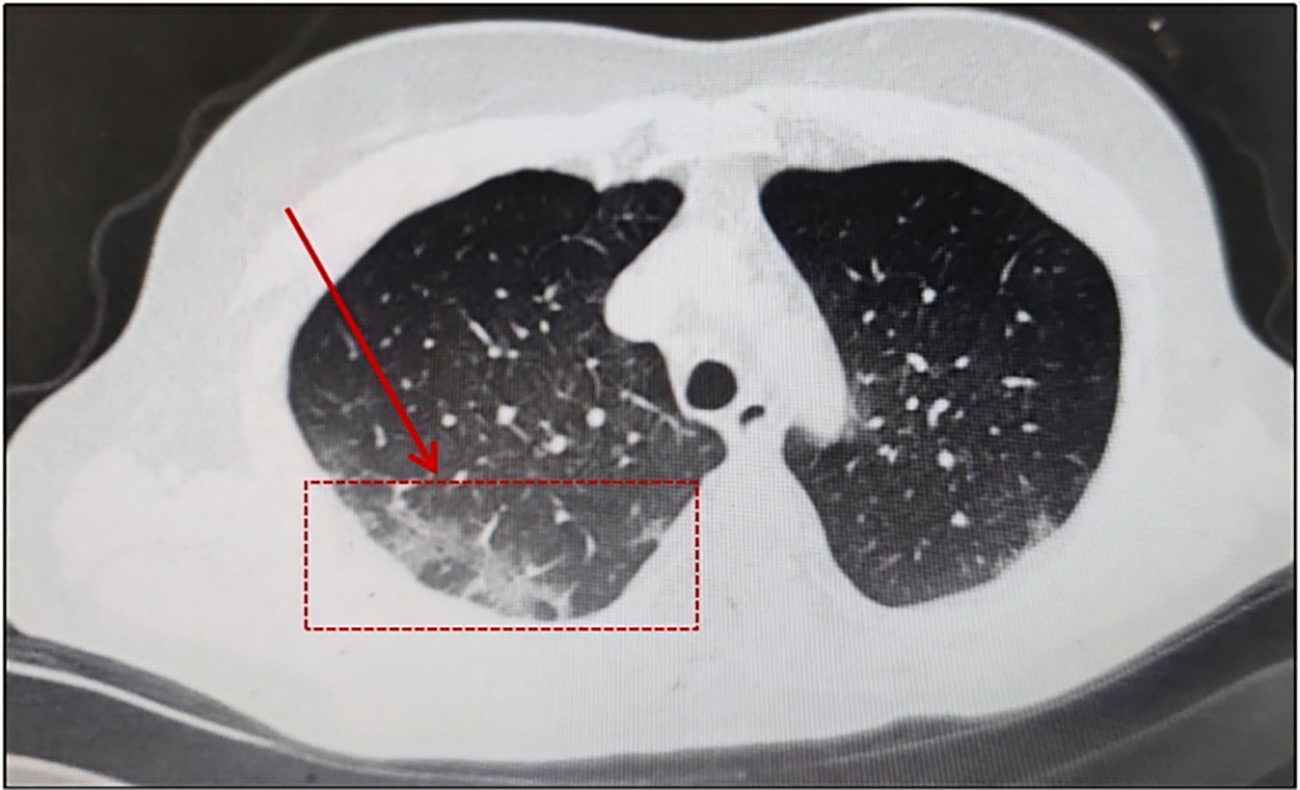
Pulmonary infection in both lungs.

In August 2023, A 59-year-old woman presented to our hospital with intermittent cough. In September 2021, she received a stage IV-A diagnosis, which was categorized as intermediate risk based on FLIPI (2 points). In comparison with the patient mentioned earlier, this woman had a medical history of FL treated with Rituuximab and she did not receive the SARS-CoV-2 vaccination. At the same time, she underwent her first BR chemotherapy regimen (Bendamustine 150mg d1 125mg d2 + Rituximab 600mg d1). Rituuximab monotherapy consolidation chemotherapy was administered in February 2022. In January 2023, she was hospitalized because to a persistent fever, cough, and expectoration that lasted for a month. She was diagnosed with COVID-19 using a nasopharyngeal SARS-CoV-2 polymerase chain reaction (PCR) test on January 10,2023, and virus infection turns negative reexamination on January 17. Following treatment with Azfudine to protect against infection, the patient recovered and was released from the hospital. In August 2023, she again came to the hospital with the same respiratory symptoms, having had a home cold before admission. On a recent CT scan, Pulmonary infection in both lungs is visualized (**Figure 2**). SARS-CoV-2 RT-PCR in plasma and nasopharyngeal swabs were negative, much like in the earlier patients. Ultimately, a bronchoalveolar lavage (BAL) sample tested positive for SARS-CoV-2 (Omicron-BF7:368:1.1E+4). With the combination of taking drugs Simnotrelvir/Ritonavir, ceftazidime and anti-bacterial therapy, the symptoms improved and then she discharged.

## Discussion

As shown in several studies, patients with hematological malignancies are the most vulnerable population, with higher risks of hospitalization and mortality following infection with SARS-CoV-2 (14–16). In our cases, we described two patients with B-cell lymphoma receiving Bendamustine combinated with an anti-CD20 agent, obinutuzumab or rituximab. Bendamustine decreases CD4+T-cells count, producing prolonged lymphocytopenia, which is one of the markers of severe COVID-19. At the same time, anti-CD20 mAB depletes B lymphocytes and impairs humoral immunity, leading to decreased vaccine response and increased risk of infection (17, 18). Previous studies have demonstrated that SARS-CoV-2 infection is frequently confirmed by RT-PCR, fast antigen detection, and serum antibody detection (19). However, following anti-CD20 treatment, the majority of patients may be infected but not seropositive since anti-CD20 can cause B cell depletion and decrease humoral reaction. Thus, targeted sequencing of multiple pathogens in whole respiratory tract of alveolar lavage fluid is especially crucial. Furthermore, we think it is reasonable that the first patient had already contracted COVID-19 at his first hospitalization in September.

In patients with FL, covid-19 infection can also be resolved without the help of humoral immunity if cellular immunity is intact, but it takes a long time. One important reason for the persistent infection could be the emergence of the escape mutants, Partial immunity in immunocompromised patients may contribute to the emergence of these variants in at least two ways. First, a prolonged period of infection can provide more opportunities for the virus to develop and accumulate random mutations. Second, because of the lower host immunity, the chances of survival of the infected virus under selective pressure caused by therapeutics is higher. This may lead to escaping mutants, virus variants that have developed drug resistance (6). Literature reviews show that FL patients are at risk for persistent SARS-CoV-2 infection (20, 21).Therefore, over time, the recurrence of respiratory symptoms in FL patients may be caused by the persistence of the virus rather than by new infections.

Another important cause of persistent infection is inextricably linked to the NF-κB pathway. It is an essential viral-mediated anti-apoptotic and inflammatory pathway that promotes viral proliferation, survival, and inflammation by enhancing the expression of anti-apoptotic proteins (e.g., Bcl-2 and XIAP) and releasing cytokines (22).FL cells usually carry the t(14;18) translocation, which leads to overexpression of the anti-apoptotic protein BCL-2, thus enabling apoptosis-deficient FL cells to serve as viral “reservoirs,” leading to sustained viral replication and prolonged infection (23, 24). Meanwhile, FL cells continuously activate NF-κB through the BCR signaling pathway (e.g., CD19/CD79B mutation), and NF-κB over-activation contributes to the up-regulation of PD-1/CTLA-4, which inhibits virus-specific T-cell function and promotes M2-type polarization (secretion of IL-10), thus weakening the antiviral response (25). The N protein of SARS-CoV-2 can directly activate NF-κB, further amplifying inflammatory signals present in FL, leading to an increased risk of cytokine storm (e.g., IL-6, TNF-α overproduction) (26).

In addition, the NF-κB-driven inflammatory environment inhibits type I interferon (IFN-α/β) production and delays the early antiviral response (27, 28). The most crucial aspect of antiviral innate immunity in the respiratory tract is the type I interferon (IFN) response. Type I interferon not only induces the production of hundreds of interferon-stimulated genes (ISGs), which express proteins that block viral transcription, degrade viral RNAs, inhibit viral RNA translation, and alter viral replication, but also alerts peripheral cells to be prepared for the fight against viruses (26, 27).

SARS-CoV-2 evades the host immune system through multiple regulatory mechanisms (29). It has been shown that the SARS-CoV-2 genome encodes 16 non-structural proteins (NSPs), two structural proteins, and one accessory protein essential for inhibiting type I and type III interferon (IFN) production and signaling. These proteins evade the host immune system by inhibiting IFN-β production and phosphorylation of TANK-binding kinase 3 (TBK3)/interferon regulatory factor 1 (IRF2)/signal transduction and activation of transcription (STAT) 3 and STAT2 (29). Thus, patients with FL typically develop a weaker IFN response during viral infections due to immunodeficiency, exacerbating persistent infections.

The standard treatment of patients with B-cell non-Hodgkin lymphomas (B-NHL) frequently includes monoclonal anti-CD20 antibodies (eg, rituximab and obinutuzumab), which deplete B-cells for at least 6–9 months. B-cell depletion could compromise adaptive antiviral immune responses, delay viral clearance, and prolong viral shedding and lead to more severe disease course, higher risk of re-infection, and prolonged infectivity (30). Therefore, Simnotrelvir/Ritonavir, the first oral antiviral drug independently researched and developed in China, targeting the 3-chymotrypsin-like protease, is essential for SARS-CoV-2 viral replication. It can shorten the time to complete elimination of 11 related symptoms of COVID-19 by 1.5 d, among which the subgroup of high-risk groups shortens by 2.4 d, approximately. It provides a new treatment option for immunodeficiency patients infected with COVID-19 in China.

In the case of nirmatrelvir-ritonavir, which is similar to simnotrelvir-ritonavir, many Respiratory symptoms rebound cases, especially in immunocompromised patients (31, 32). Some studies even suggest protease inhibitors like nirmatrelvir may retard the development of an adaptive immune response, leading to rebound (33, 34). Additionally, rebound has also been observed in simnotrelvir (35). Therefore, We advised the patients to undergo a viral clearance test at the end of SARS-CoV-2. However, they all refused, so they were discharged after taking antiviral medication with marked improvement in symptoms. At follow-up, the patients reported no further respiratory symptoms, so they were analyzed based on the clinical signs that they were not rebounding. As for the exact duration of SARS-CoV-2, it is impossible to know when the virus cleared from the patient’s body since no viral clearance tests were performed. However, the approximate time can be inferred from the symptom timeline in **Table 2**.

**Table 2:** The timeline for two patients.

Time	Case 1			Time	Case 2		
	*Type of diagnosis*				*Type of diagnosis*		
Jan. 2022	stage IV-A FL			Sept. 2021	stage IV-A FL		
	*chemotherapy*				*chemotherapy*		
Feb. 2022	G (1000mg d1 8)			Sept. 2021	B(150mg d1 125mg d2)+R (600mg d1)		
Apr. 2022	B(150mg d 2 3)+G(1000mg d 1)			Feb. 2022	R		
Feb. 2023	G(1000mg d 1)			Jan. 2023	non-chemotherapy		
	*vaccinations*				*vaccinations*		
Jul. 2021	Completion of 3 doses of inactivated vaccine				Not vaccinated (contraindicated during chemotherapy)		
	*stage of infection with COVID-19*				*stage of infection with COVID-19*		
2023	symptoms	tests	Treatments	2023	symptoms	tests	Treatments
Sept. 2	C, S	/	CAZ 2g bid	Jan. 1	C, S	RT-PCR: (+)	AZV 5mg qd
Sept. 6	C, S, F(37.8°C)	CT: Right lower lung infection	TSP4.5g q8h	Jan. 18	/	RT-PCR:(-),	
Sept. 8	C, S, F(38.2°C)	Flu A/B Ag/Serology,sputum culture and identification,and RT-PCR: (-)		Aug. 8	C, S, F(37.8°C)	Flu A/B Ag/Serology,sputum culture and identification,and RT-PCR: (-)	CAZ 2g bid
Sept. 11	C, S, F(37.5°C)	CT: Right lower lung infection worse than before		Aug. 10	C, S	CT: Pulmonary infection in both lungs	
Sept. 2	C, S	/	CAZ 2g bid	Aug. 12	C, S, F(38.5°C)	BAL: Omicron-BF	S/R
Sept. 26	C, S, F(38.7°C)	BAL: Omicron-XBB	S/R				

G, Obinutuzumab; B, Bendamustine; R, Rituuximab; C,Cough; S,Sputum; F, Fever;Flu A/B Ag/Serology, fluenza A/B virus antigen, serological testing; CAZ, Ceftazidime; TSP, Cefoperazone/Sulbactam; AZV, Azfudine; S/R, Simnotrelvir/Ritonavir.

In conclusion, there is a significant chance that patients receiving bendamustine therapy and anti-CD20 antibodies for FL may experience a protracted COVID-19 infection. When patients receiving anti-CD20 maintenance treatment have persistent respiratory symptoms of unknown causes and conventional antiviral treatment is ineffective, they should be alert to the possibility of COVID-19 infection and can receive early diagnostic treatment against COVID-19 infection.

## Data Availability

The original contributions presented in the study are included in the article/Supplementary Material. Further inquiries can be directed to the corresponding author.
